# An insight into the functional genomics and species classification of *Eudiplozoon nipponicum* (Monogenea, Diplozoidae), a haematophagous parasite of the common carp *Cyprinus carpio*

**DOI:** 10.1186/s12864-023-09461-8

**Published:** 2023-06-29

**Authors:** Jiří Vorel, Nikol Kmentová, Christoph Hahn, Petr Bureš, Martin Kašný

**Affiliations:** 1 Department of Botany and Zoology, Faculty of Science, Masaryk University, Kotlářská 2, Brno, 611 37 Czech Republic; 2 Research Group Zoology: Biodiversity and Toxicology, Centre for Environmental Sciences, Hasselt University, Agoralaan Gebouw D, Diepenbeek, B-3590 Belgium; 3 Institute of Biology, University of Graz, Universitätsplatz 2, Graz, A-8010 Austria

**Keywords:** Helminths, Monogenea, Genome, Mitochondrial genome, Assembly, Annotation, Host–parasite interaction, Sequencing, Illumina, Nanopore

## Abstract

**Background:**

Monogenea (Platyhelminthes, Neodermata) are the most species-rich class within the Neodermata superclass of primarily fish parasites. Despite their economic and ecological importance, monogenean research tends to focus on their morphological, phylogenetic, and population characteristics, while comprehensive omics analyses aimed at describing functionally important molecules are few and far between. We present a molecular characterisation of monogenean representative *Eudiplozoon nipponicum*, an obligate haematophagous parasite infecting the gills of the common carp. We report its nuclear and mitochondrial genomes, present a functional annotation of protein molecules relevant to the molecular and biochemical aspect of physiological processes involved in interactions with the fish hosts, and re-examinate the taxonomic position of *Eudiplozoon* species within the Diplozoidae family.

**Results:**

We have generated 50.81 Gbp of raw sequencing data (Illumina and Oxford Nanopore reads), bioinformatically processed, and de novo assembled them into a genome draft 0.94 Gbp long, consisting of 21,044 contigs (N50 = 87 kbp). The final assembly represents 57% of the estimated total genome size (~ 1.64 Gbp), whereby repetitive and low-complexity regions account for ~ 64% of the assembled length. In total, 36,626 predicted genes encode 33,031 proteins and homology-based annotation of protein-coding genes (PCGs) and proteins characterises 14,785 (44.76%) molecules. We have detected significant representation of functional proteins and known molecular functions. The numbers of peptidases and inhibitors (579 proteins), characterised GO terms (16,016 unique assigned GO terms), and identified KEGG Orthology (4,315 proteins) acting in 378 KEGG pathways demonstrate the variety of mechanisms by which the parasite interacts with hosts on a macromolecular level (immunomodulation, feeding, and development). Comparison between the newly assembled *E. nipponicum* mitochondrial genome (length of 17,038 bp) and other diplozoid monogeneans confirms the existence of two distinct *Eudiplozoon* species infecting different fish hosts: *Cyprinus carpio* and *Carassius* spp.

**Conclusions:**

Although the amount of sequencing data and characterised molecules of monogenean parasites has recently increased, a better insight into their molecular biology is needed. The *E. nipponicum* nuclear genome presented here, currently the largest described genome of any monogenean parasite, represents a milestone in the study of monogeneans and their molecules but further omics research is needed to understand these parasites’ biological nature.

**Supplementary Information:**

The online version contains supplementary material available at 10.1186/s12864-023-09461-8.

## Background

Current comprehensive genomic resources for parasitic helminths tend to focus on the causative agents of severe human and animal diseases that have a major impact on socioeconomic development. Although several hundred thousand species of parasitic helminths are believed to exist [1], only a handful of their genomes are accessible in public databases. For example, the current version of the WormBase ParaSite database (WBPS18) includes 240 genomes, 177 of which belong to nematodes and 63 to platyhelminths [2]. Although in the past few years we have witnessed a dramatic increase in publicly available helminth genomes (to compare: just over 30 genomes were available in 2014 [3]) and associated omics research, genomics of parasitic helminths is still a relatively neglected area of biological research [2, 4, 5]. This is unfortunate, especially given that a better understanding of the molecular and biochemical nature of parasitic helminths could reveal mechanisms which drive evolutionary host–parasite interactions and the emergence of drug resistance [6–9].

Monogeneans are mainly ectoparasites of freshwater and marine bony fishes, with only a low number of species infecting cartilaginous fish or semi-aquatic tetrapods: only about 250 monogenean species that parasite amphibians and chelonians are known at present [10]. Compared to other groups of parasitic flatworms, their lifecycles are direct and short, and they are believed to be the most host-specific parasites among flatworms [11, 12]. Infestation by monogeneans can cause in the fish hosts serious and often lethal diseases, leading to significant economic losses in commercial fish-breeding stocks, especially those with a high stocking density [13, 14]. As demonstrated by a number of studies [15–18], the Monogenea class is, in terms of its molecular, structural, and functional characteristics, probably the most diverse group within the obligate parasitic superclass Neodermata, and a better understanding of monogeneans is key to deciphering the evolution of parasitism in flatworms. Monogeneans tend to be studied in terms of phylogenetics [19, 20] and population characteristics [21–23]. The number of comprehensive molecular analyses of monogeneans is increasing rather slowly: since the last summarisation in late 2020 [24], only one new experimental omics work has been published [25]. This most recent work presents de novo assembled transcriptomes of two monopisthocotyleans: *Scutogyrus longicornis* (family Ancyrocephalidae; 25,696 predicted proteins), which parasitises the Nile tilapia *Oreochromis niloticus*, and *Rhabdosynochus viridisi* (family Diplectanidae; 47,187 predicted proteins), which infects the white snook *Centropomus viridis*.

*Eudiplozoon nipponicum*, the organism that is the focus of the current study, is a haematophagous ectoparasite belonging to the family Diplozoidae, which had since its introduction from East Asia become a common parasite of the European fauna [26]. This oviparous helminth with a unique lifecycle inhabits the gills of the common carp *Cyprinus carpio*. The first larval stage, oncomiracidium, after hatching from an oval-shaped egg actively moves in the water environment and searches for a host. After attaching itself to the host’s gills, oncomiracidium develops into the next stage, diporpa, a sexually immature larva. Later, two diporpae permanently fuse to form the juvenile stage, which matures into an adult X-shaped individual [27, 28]. Infection by *E. nipponicum* does not per se lead to a premature death of the fish host, but the parasite’s blood-feeding strategy – which involves a mechanical disruption of host gill tissue to ensure continuous blood flow – causes anaemia and facilitates secondary bacterial and viral infections [14]. All this takes its toll especially on heavily infested younger fish.

Detailed knowledge of the molecular principles of *E. nipponicum* biology and the parasite’s functional proteins is still limited (as reviewed in [24]), whereby existing knowledge is based mainly on the description of peptidases (cathepsins B, D, L1 and L3) [29, 30] and their inhibitors, namely Kunizt-type inhibitor [31], serpin [32], and cystatin (stefin) [33]. Further comprehensive studies targeted the transcriptome and secretome, set of excretory–secretory proteins [24], and tissue-specific proteome [34] of adult worms. Currently, two mitochondrial genomes are available for members of the genus *Eudiplozoon*. The first belongs to an unspecified species *Eudiplozoon* sp. (14,334 base pairs [bp] in length, fish host *Carassius auratus*, East China origin, NCBI GenBank accession number MG458328.1) [35], the second to *E. nipponicum* specifically (17,328 bp in length, unknown fish host and origin, NCBI GenBank accession number NC_061193.1, unpublished).

Despite advances in monogenean research, the taxonomy of monogeneans remains convoluted and disputed. Recently, the species status of *E. nipponicum* was reassessed as being specific to the host *Carassius* sp., and a new species was described, namely *Eudiplozoon kamegaii*, which infects *C. carpio* [36].

This study presents the first draft of *E. nipponicum* genome assembly, with in silico annotation and characterisation of functional protein molecules and biochemical pathways involved in host–parasite interaction. This genome draft should serve as a robust data platform for future in-depth analyses addressing molecular description of the highly diverse monogenean flatworms. With a previously described transcriptome, excretory–secretory and tissue-specific proteomes, and several characterised peptidases and inhibitors, *E. nipponicum* is the most studied monogenean on the level of functional genomics to date, and information pertaining to this parasite is fast developing into a promising model system with a huge potential for functional genomics studies. Additionally, we have used the mitochondrial genome of *E. nipponicum* presented here re-examine the species diversity of *Eudiplozoon* spp. in relation to the host species and compared it with other representatives of Diplozoidae.

## Results

### Characteristics of genome assembly and predicted genes, estimation of genome size

We have sequenced 329,260,590 Illumina reads (164,630,295 pairs) and 2,781,863 Oxford Nanopore Technologies (ONT) reads (N50 length 7,552.0 bp), which represents a total yield of 50.81 raw Gbp. After data processing (before the assembly), 36.36 Gbp were divided between 232,634,716 Illumina reads (read length 80–251 bp) and 2,287,049 ONT reads (N50 length 8,232.0 bp). Complete statistics pertaining to the raw and processed reads are summarised in (Table 1) for Illumina reads and (Table 2) for ONT reads.

**Table 1:** Complete statistics of Illumina raw and processed (pre-assembly) reads

**Illumina raw reads**			
**Library name**	**No. of reads**	**No. of bases**	**Read length**
A7KL0	21,566,020	5,413,071,020	251 bp
A72DD	26,548,906	6,335,791,067	251 bp
C4VFYACXX	68,851,956	6,954,047,556	101 bp
C5KL9ANXX	78,887,700	9,860,962,500	125 bp
C841DACXX	133,406,008	13,474,006,808	101 bp
**Illumina processed reads**			
**Library name**	**No. of reads**	**No. of bases**	**Average read length**
A7KL0	16,160,827	3,716,083,176	223 bp
A72DD	17,203,918	4,024,933,621	234 bp
C4VFYACXX	41,793,309	4,146,772,393	99 bp
C5KL9ANXX	50,820,258	6,197,086,878	122 bp
C841DACXX	106,656,404	10,554,928,161	99 bp

**Table 2:** Complete statistics about Oxford Nanopore Technologies raw and processed (pre-assembly) reads

Metric	Raw reads	Processed reads
No. of reads	2,781,863	2,287,049
Total bases	8,772,164,918	7,723,898,588
Mean read length	3,153.3 bp	3,377.2 bp
Median read length	1,315.0 bp	1,386.0 bp
Mean read quality	10.6	12.9
Median read quality	11.4	13.1
Read length N50	7,552.0 bp	8,231.0 bp
STDEV read length	6,326.1 bp	6,660.4 bp

Processed (by quality trimming and filtering, error correction, and de-duplication) and contamination-free Illumina reads were used to estimate the genome size. Bioinformatic prediction based on k-mer occurrence estimated the genome size at 1.63 Gbp ± 198.66 Mbp; this assessment was supported by an experimental measurement by flow cytometry (1C = 1.65 Gbp ± 103.06 Mbp). A combination of Flye and MaSuRCA assemblers, with subsequent processing and filtering on the level of contigs, yielded the final draft of the *E. nipponicum* genome, which is 0.94 Gbp long and contains 21,044 contigs (Table 3). Compared to the estimated genome size, which was over 1.64 Gbp (the mean of k-mer-based prediction and flow cytometry measurement), this amounts to a completeness level of over 57% of the assembly. The assembly does not resolve the chromosome level (seven pairs of telocentric chromosomes) [37]. Complete statistics on the final version of the *E. nipponicum* genome, including the intermediate steps, are summarised in (Additional file 1: Table S1).

**Table 3:** Basic statistics of the final draft of the *Eudiplozoon nipponicum* genome

Basic statistics	
No. of contigs	21,044
Total genome length	939,802,929 bp
Masked bases	601,146,580 bp (63.97%)
Longest contig	557,136 bp
GC content	34.97%
No. of unidentified (N) bases	0
N50	87,067 bp
L50	3,219 bp
BUSCO analysis (954 searched groups)	C: 40.1% (S: 37.1%, D: 3.0%), F: 13.9%, M: 46.0%
No. of genes	36,626
No. of PCGs	33,031

Repetitive elements and low-complexity regions form a considerable part of the *E. nipponicum* genome: 63.97% of the original genome length was masked (Additional file 2: Table S2). We found in the *E. nipponicum* genome a total of 609 repeat families. Uncharacterised repeats, where specific class is missing, are the primary source of all masked bases (~ 30.2%). They are followed by class I transposable elements (retrotransposons), such as LINE/Penelope (~ 9.3%), LTR/Gypsy (~ 7.7%), LINE/RTE-BovB (~ 5.6%), and LINE/CR1 (~ 2.9%), and by class II transposable elements (DNA transposons), such as DNA/CMC-EnSpm (~ 2.7%) and RC/Helitron (~ 1.1%). Simple sequence repeats (microsatellites) amount to ~ 2.4% of all masked bases.

A total of 36,626 genes were predicted in the *E. nipponicum* genome, 33,031 (90.18%) of which are protein-coding. On average, a typical *E. nipponicum* PCG 6,550 bp long consists of three exons (with average exon length 279 bp) and two introns (with average intron length 3,098 bp) and encodes an mRNA transcript 798 bp long. The total length of exons represents 2.80% of the assembled genome (26,354,862 bp). Completeness of the coding regions in the genome assembly was evaluated by mapping *E. nipponicum* RNA-seq raw reads (generated in a previous study [24]) to genomic contigs. A total of 83.19% of paired-end RNA-seq raw reads were mapped to the genome. For 13,473 (44.10%) of proteins predicted from the genome, we found homologues with published *E. nipponicum* translated transcripts [24].

### A summary of homology-based annotation of mRNA transcripts and proteins

**Fig. 1:**
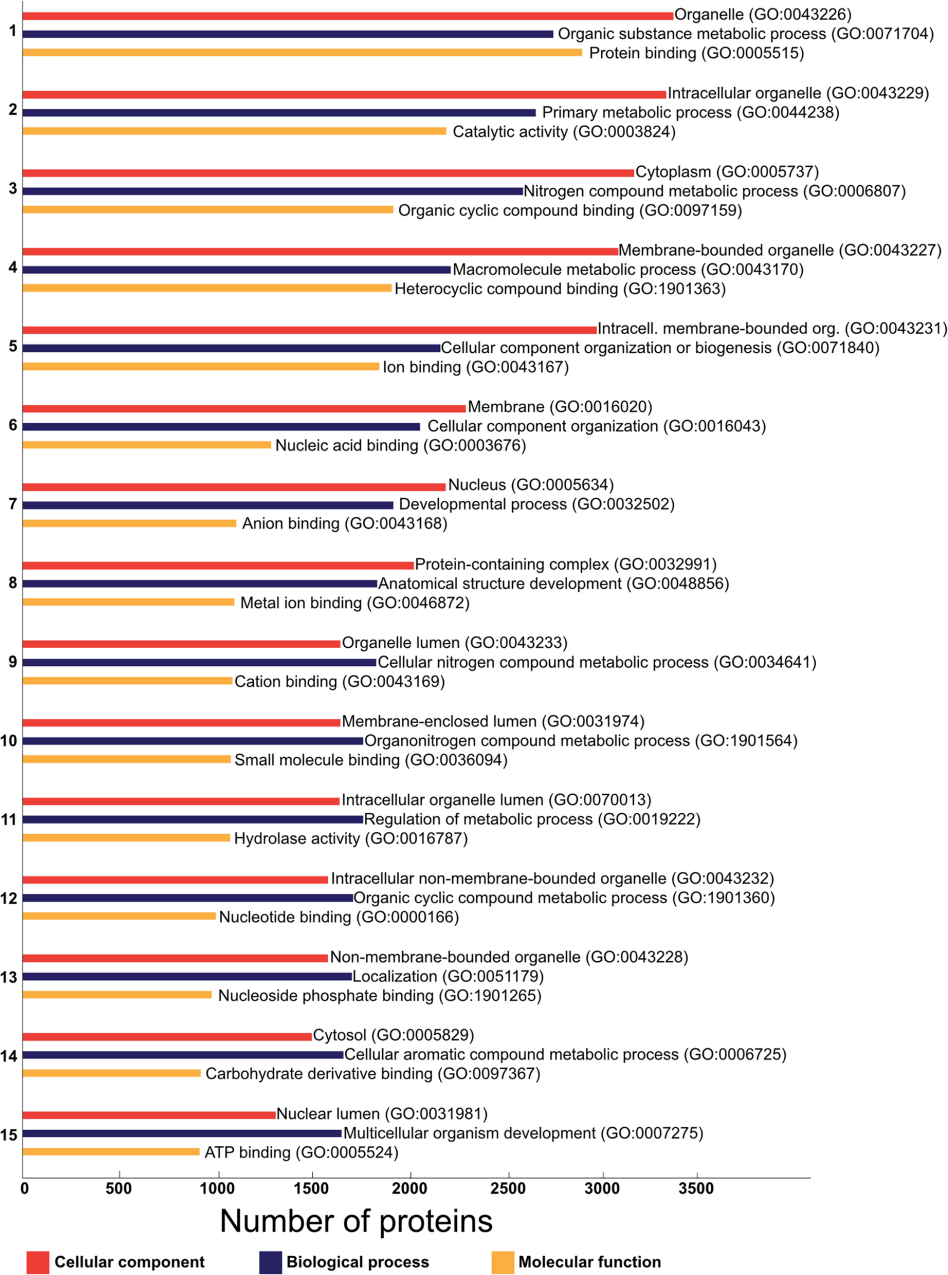
The most abundant GO terms. Top 15 GO terms in each category quantified according to the number of included proteins (axis x) and sorted in descending order (axis y)

We have aligned a total of 33,031 PCGs and their translated proteins to several protein databases and one nucleotide database to predict their functions. Homology-based annotation yielded at least one significant hit for 14,785 (44.76%) *E. nipponicum* PCGs and proteins (Table 4; Additional file 3: Table S3) and 16,016 unique gene ontology (GO) terms were assigned to 7,460 proteins (22.59%). Category ‘biological processes’ was the most represented based on the number of GO terms (*n* = 11,331, 70.75%); it was followed by categories ‘molecular function’ (*n* = 2,662, 16.63%) and ‘cellular component’ (*n* = 1,653, 1.03%). The numbers of individual proteins included in each GO term were calculated, and the most abundant GO terms (in each main category) are presented in (Fig. 1). We have observed a high number of proteins related to binding functions (e.g., protein binding, *n* = 2,841; organic cyclic compound binding, *n* = 1,882; nucleic acid binding, *n* = 1,261), which act in many essential cellular processes. Abundant GO term hydrolase activity (GO:0016787, *n* = 1,054) includes proteins catalysing the hydrolysis of various bonds, including various peptidases acting on the host-parasite interface (e.g., anticoagulation, digestion of host blood, modulation of the immune system). Extracellular vesicles are secreted structures which transfer a range of molecules affecting the hosts and facilitate the parasite’s survival [38]. The representation of GO terms ‘vesicle’ (GO:0031982, *n* = 591) and child terms ‘intracellular vesicle’ (GO: 0097708, *n* = 569), ‘vesicle membrane’ (GO: 0012506, *n* = 173), ‘vesicle lumen’ (GO: 0031983, *n* = 90), and ‘extracellular vesicle’ (GO:1,903,561, *n* = 27) indicate a considerable capacity for vesicle biogenesis.

**Table 4:** A summary of results from homology-based annotation

Database	Number	Representation
PCGs	33,031	Complete dataset (100%)
InterPro	12,236	37.04%
eggNOG	5,746	17.40%
MEROPS peptidases	521	1.58%
MEROPS inhibitors	58	0.18%
UniProt/UniRef100	11,416	34.56%
NCBI protein (nr)	11,395	34.50%
NCBI nucleotide (nt)	1,167	3.53%
KEGG orthology (KO)	4,315	13.06%
Proteins with GO terms	7,460	22.59%
Overall annotated	14,785	44.76%
Overall unannotated	18,246	55.24%

In line with the latest phylogenetic analysis confirming a closer relationship of the monogenean subclass Polyopisthocotylea to trematodes than to cestodes [18], we observed that most *E. nipponicum* proteins are homologues (based on annotation in the UniProt/UniRef100 protein database) to monogenean representative *Protopolystoma xenopodis* (*n* = 2,360), followed by trematodes, such as *Paragonimus westermani* (*n* = 608), *Schistosoma japonicum* (*n* = 462), *Echinostoma caproni* (*n* = 429), and cestode *Schistocephalus solidus* (*n* = 410) (Table 5).

**Table 5:** Top 20 organisms with the most significant number of protein homologues, deposited in the UniProt/UniRef100 database, to *Eudiplozoon nipponicum*

Organism	Class	No. of homologues
*Protopolystoma xenopodis*	Monogenea	2,360
*Paragonimus westermani*	Trematoda	608
*Schistosoma japonicum*	Trematoda	462
*Echinostoma caproni*	Trematoda	429
*Schistocephalus solidus*	Cestoda	410
*Fasciolopsis buski*	Trematoda	405
*Clonorchis sinensis*	Trematoda	391
*Opisthorchis viverrini*	Trematoda	322
*Paragonimus heterotremus*	Trematoda	317
*Fasciola hepatica*	Trematoda	316
*Trichobilharzia regenti*	Trematoda	307
*Fasciola gigantica*	Trematoda	294
*Opisthorchis felineus*	Trematoda	292
*Paragonimus skrjabini miyazakii*	Trematoda	282
*Schistosoma mansoni*	Trematoda	259
*Mesocestoides corti*	Cestoda	216
*Dibothriocephalus latus*	Cestoda	203
*Schistosoma rodhaini*	Trematoda	195
*Spirometra erinaceieuropaei*	Cestoda	193
*Schistosoma mattheei*	Trematoda	184

Annotation in KEGG databases [38] (mediated by the eggNOG-mapper annotation tool [39]) assigned 3,304 unique K numbers (functional orthologs) to 4,315 proteins (13.06%), classified 2,933 proteins (8.88%) into 378 KEGG pathways (molecular interaction, reaction, and relation networks), and linked 1,087 (*n* = 3.29%) proteins to 249 KEGG modules (functional units of gene sets). *Eudiplozoon nipponicum* lost the genetic ability to synthesise fatty acids de novo. Based on annotation in the KEGG database, KEGG pathway map ko00061 (Fatty acid biosynthesis) contains only five identified enzymes (Additional file 4: Figure S1). In particular, (*i*) acetyl-CoA carboxylase (EC 6.4.1.2), which starts the entire pathway by carboxylation of acetyl-CoA to form malonyl-CoA. In the next step, the malonate is transferred to acyl carrier protein by (*ii*) [acyl-carrier-protein] S-malonyl transferase (EC 2.3.1.39), while (*iii*) 3-oxoacyl-[acyl-carrier-protein] synthase II (EC 2.3.1.179) and (*iv*) trans-2-enoyl-CoA reductase (EC 1.3.1.38) participate in the process of elongation of fatty acid chain. And finally, (*v*) long-chain acyl-CoA ligase (EC 6.2.1.3) catalyses the conversion of fatty acids to their active form.

The eggNOG database [40] classified 1,722 proteins (5.21%) as enzymes with a numerical classification (EC numbers), whereby the most abundant main classes were oxidoreductases (*n* = 182,

10.60%), transferases (*n* = 778, 45.18%), hydrolases (*n* = 534, 31.01%), lyases (*n* = 73, 4.24%), isomerases (*n* = 56, 3.25%), and ligases (*n* = 99, 5.75%). InterPro [41] analysis characterised 12,236 (37.04%) *E. nipponicum* proteins with significant numbers of homologues in databases PANTHER (*n* = 9,655), Gene3D (*n* = 7,697), Pfam (*n* = 7,166), SUPERFAMILY (*n* = 6,934), ProSiteProfiles (*n* = 3,805), SMART (*n* = 2,883), CDD (*n* = 1,859), ProSitePatterns (*n* = 1,599), FunFam (*n* = 1,500), PRINTS (*n* = 1,245), TIGRFAM (*n* = 230), and SFLD (*n* = 7). All in all, 7,166 *E. nipponicum* proteins with identified Pfam domains were distributed among 2,763 unique domains. Based on the frequency of occurrence, the 15 most common domains are summarised in (Table 6).

**Table 6:** Top 15 Pfam domains

Accession	No. of proteins	Name	Function
PF00069	148	Protein kinase domain	Regulatory and signalling functions [42]
PF00076	118	RNA recognition motif	RNA processing and modification, affecting gene expression [43]
PF00096	113	Zinc finger, C2H2 type	Transcription factor, regulation of immune response, cell differentiation and development [44]
PF00250	110	Forkhead domain	Transcription factor affecting development [45]
PF00400	98	WD domain, G-beta repeat	Protein–protein binding interactions, various cellular functions [46]
PF00271	63	Helicase conserved C-terminal domain (DEAD/H)	Participates in RNA metabolism, gene expression and immune response [47]
PF13499	55	EF-hand domain pair	Regulatory and structural functions, calcium-binding proteins [48]
PF12796	53	Ankyrin repeats	Protein–protein interactions, diverse cellular functions [49]
PF00188	53	Cysteine-rich secretory protein family (CAP)	Various physiological functions, reproduction [50, 51]
PF00001	51	7 transmembrane receptor (rhodopsin family)	Transmembrane proteins with a high functional diversity [52]
PF04857	50	CAF1 family ribonuclease	mRNA processing, deadenylation [53]
PF00046	50	Homeodomain	DNA binding activity with a wide variety of biological functions [54, 55]
PF00125	44	Core histone H2A/H2B/H3/H4	Structural functions [56]
PF00520	43	Ion transport protein	Sodium, potassium, and calcium ion channels [57]
PF00270	43	DEAD/DEAH box helicase	Participating in RNA metabolism [58]

### Peptidases and inhibitors

**Fig. 2:**
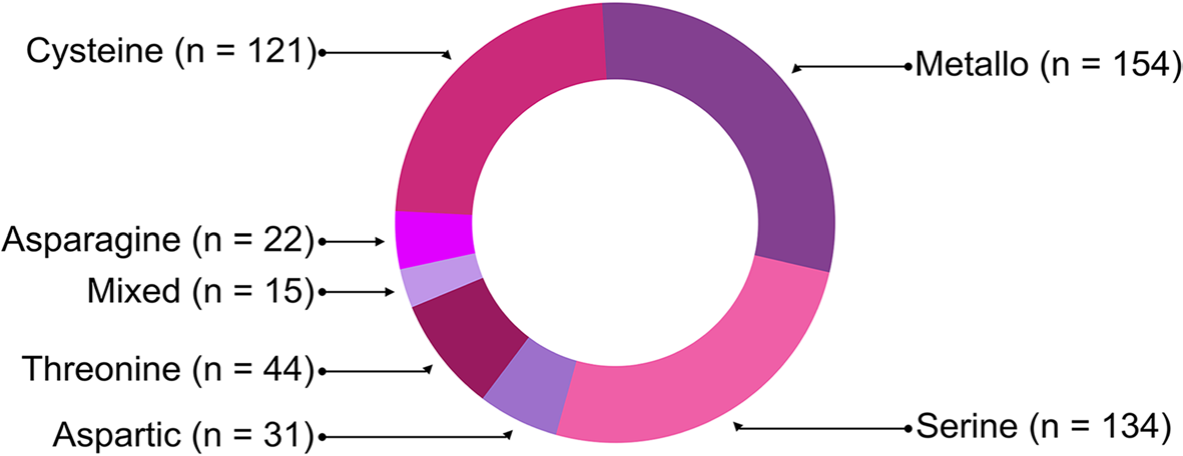
*Eudiplozoon nipponicum* peptidases. Distribution of individual peptidase catalytic types based on the MEROPS database

A total of 579 (1.75%) *E. nipponicum* proteins were aligned with records deposited in the MEROPS database (521 peptidases and 58 inhibitors) [59]. *Eudiplozoon nipponicum* protein sequences annotated as ‘unassigned peptidase inhibitors’, ‘unassigned peptidases’, or ‘non-peptidase homologues’ were excluded from further analyses. Peptidases of the metallo-catalytic type form the most numerous group in the proteolytic apparatus of *E. nipponicum* (*n* = 154, 29.56%), followed by serine (*n* = 134, 25.72%) and cysteine (*n* = 121, 23.22%) peptidases in the second and third place, respectively (Fig. 2). Peptidases of an unknown catalytic type (U) were labelled ‘unassigned peptidases’ and excluded from further analyses. MEROPS search revealed no glutamic peptidases at all. Protein homologues to peptidases (*n* = 521) were divided into 68 families (Table 7; Additional file 5: Table S4). Their number (*n* = 521) is nine times higher than the number of peptidase inhibitors (*n* = 58), which are divided into 16 families (Table 8).

**Table 7:** Top 10 most abundant peptidase families revealed by annotation in the MEROPS database

Catalytic type	Family	Proteins	Type
Serine	S1	55	Chymotrypsin
Threonine	T1	44	Proteasome
Aspartic	A1	29	Pepsin
Cysteine	C1	23	Papain
Serine	S33	20	Prolyl aminopeptidase
Cysteine	C2	19	Calpain
Cysteine	C19	19	Ubiquitin-specific protease
Metallo	M1	17	Aminopeptidase N
Metallo	M2	15	Angiotensin-converting enzyme peptidase
Metallo	M24	15	Methionyl aminopeptidase 1

**Table 8:** A list of peptidase inhibitors families based on the annotation in the MEROPS database

Family	Proteins	Type
I17	13	WAP-type family (secretory leukocyte peptidase inhibitor)
I2	11	Kunitz-BPTI family (aprotinin)
I25	5	Cystatin type inhibitor
I63	4	Pro-eosinophil major basic protein
I19	4	Pacifastin family (peptidase inhibitor LMPI)
I100	4	Stanniocalcin
I31	3	Thyropin family (equistatin)
I83	2	AmFPI-1
I21	2	7B2 family (secretogranin)
I14	2	Hirudin family
I4	2	Serpin family (alpha-1-peptidase)
I1	2	Kazal family (ovomucoid)
I87	1	HflC
I44	1	Ascaris CPI family (metallocarboxypeptidase A inhibitor)
I32	1	IAP family (survivin)
I3	1	Kunitz-P family (soybean Kunitz trypsin inhibitor)

### Intra and interspecific differentiations within the Diplozoidae family

**Fig. 3:**
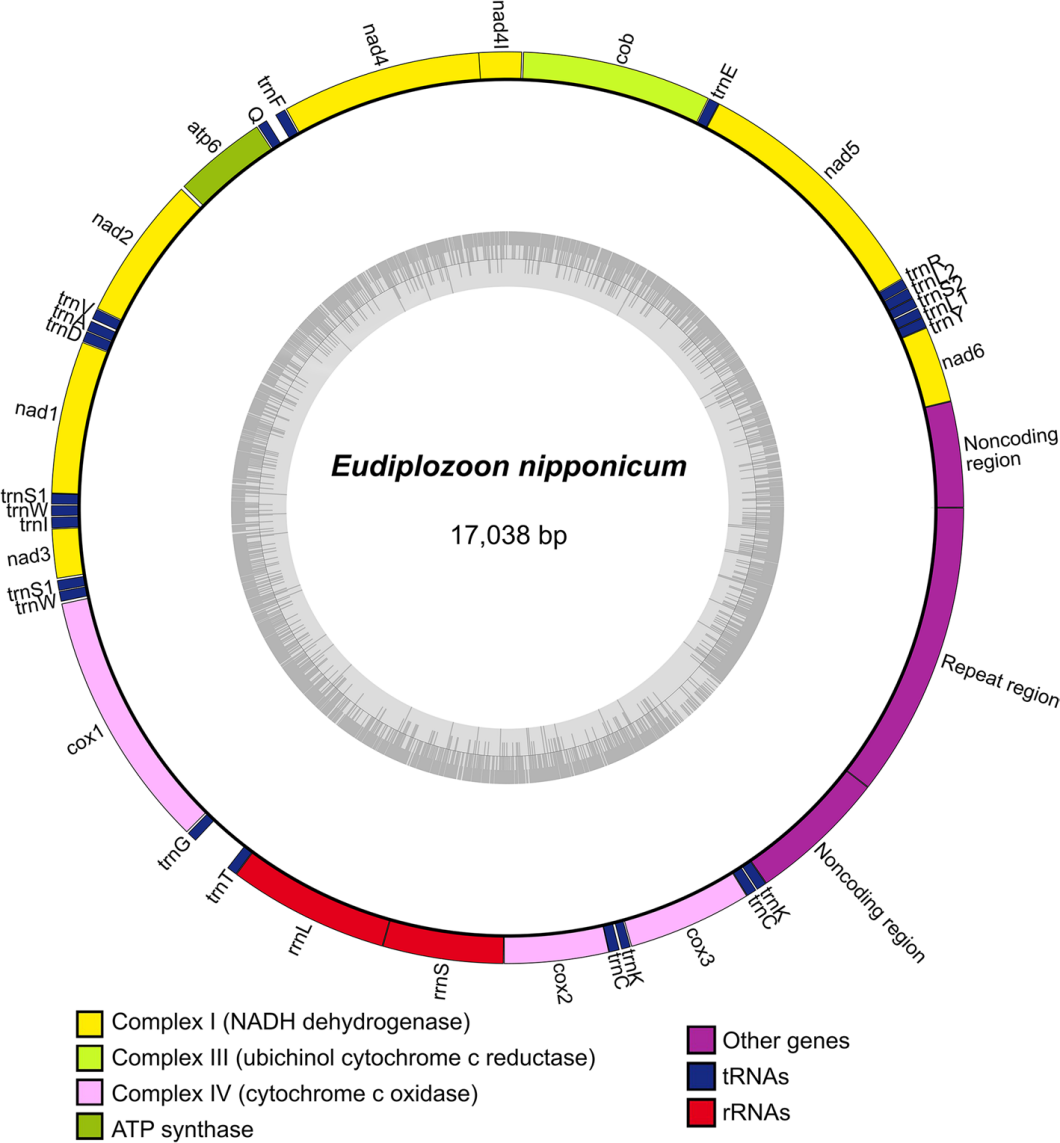
Circular map of the complete *Eudiplozoon nipponicum* mitochondrial genome. The composition of PCGs with tRNA, rRNA, and AT-rich noncoding and repeat regions in the mitochondrial genome of *E. nipponicum* (17,038 bp). The inner circle represents GC content. Light grey bars show the positions of A and T bases, and dark grey bars the position of G and C bases

The newly assembled and circularised *E. nipponicum* reference mitochondrial genome (length 17,038 bp, NCBI GenBank accession number OQ331573, Fig. 3) contains 12 PCGs (*atp8* is missing), two rRNA regions (large and small subunits), and 22 tRNA regions ordered in much the same way as in other diplozoid monogeneans (Additional file 6: Table S5). The presence of two non-coding regions (lengths 639 and 836 bp) and multiple repeats (total length 253 bp) is similar to both the previously assembled mitochondrial genomes of this parasitic species and to other representatives of polyopisthocotylean monogeneans, such as *Pseudochauhanea macrorchis*, a parasite of the pickhandle barracuda *Sphyraena jello* [60] and *Polylabris halichoeres*, which infects the bubblefin wrasse *Halichoeres nigrescens* [61]. A comparison with previously published mitochondrial genomes of polyopisthocotylean monogeneans revealed several instances of rearrangement of the tRNA genes present (*trnY*, *trnS2*, *trnL2*). In particular, in *E. nipponicum* the rearrangement took place between *nad6* and *nad5*, in *P. halichoeres* between *cox3* and *nad6*, and in *P. macrorchis* it concerned *trnY*, *trnR*, and *trnL1*. In contrast to tRNA regions, no rearrangement of PCGs have been documented in any polyopisthocotyleans.

**Fig. 4:**
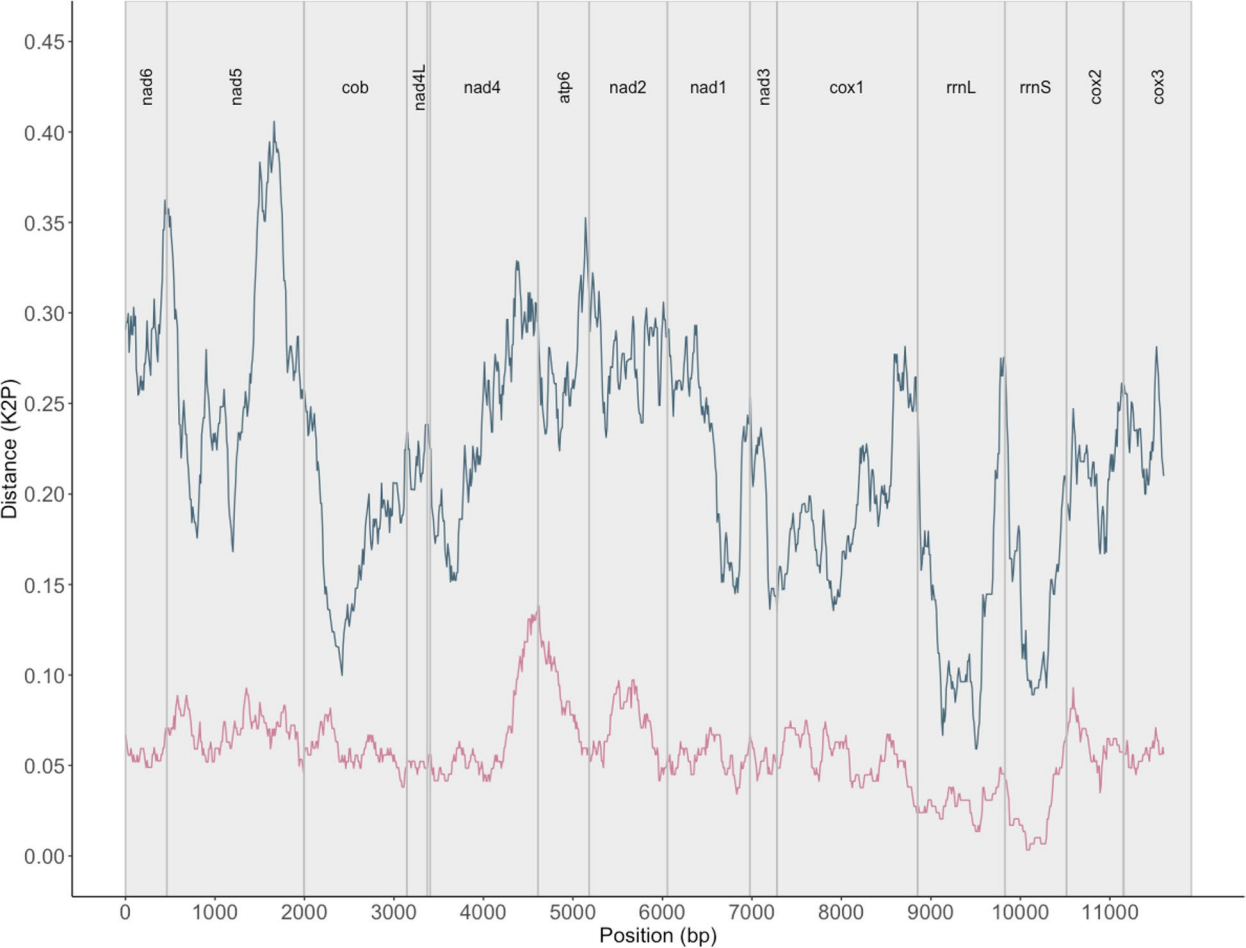
An intraspecific comparison for *Eudiplozoon* spp. mitochondrial genomes. Sliding window analyses (window size 300 bp, step size 10 bp) across the alignment of mitochondrial PCGs and rRNA regions show a pairwise comparison between *E. nipponicum* (present study – OQ331573) and *E. nipponicum* (NC_061193.1) in the pink line and between *E. nipponicum* (present study – OQ331573) and *Eudiplozoon* sp. (MG458328.1) in the blue line. The lines show the K2P distance with gene boundaries, which are indicated above the graph

**Fig. 5:**
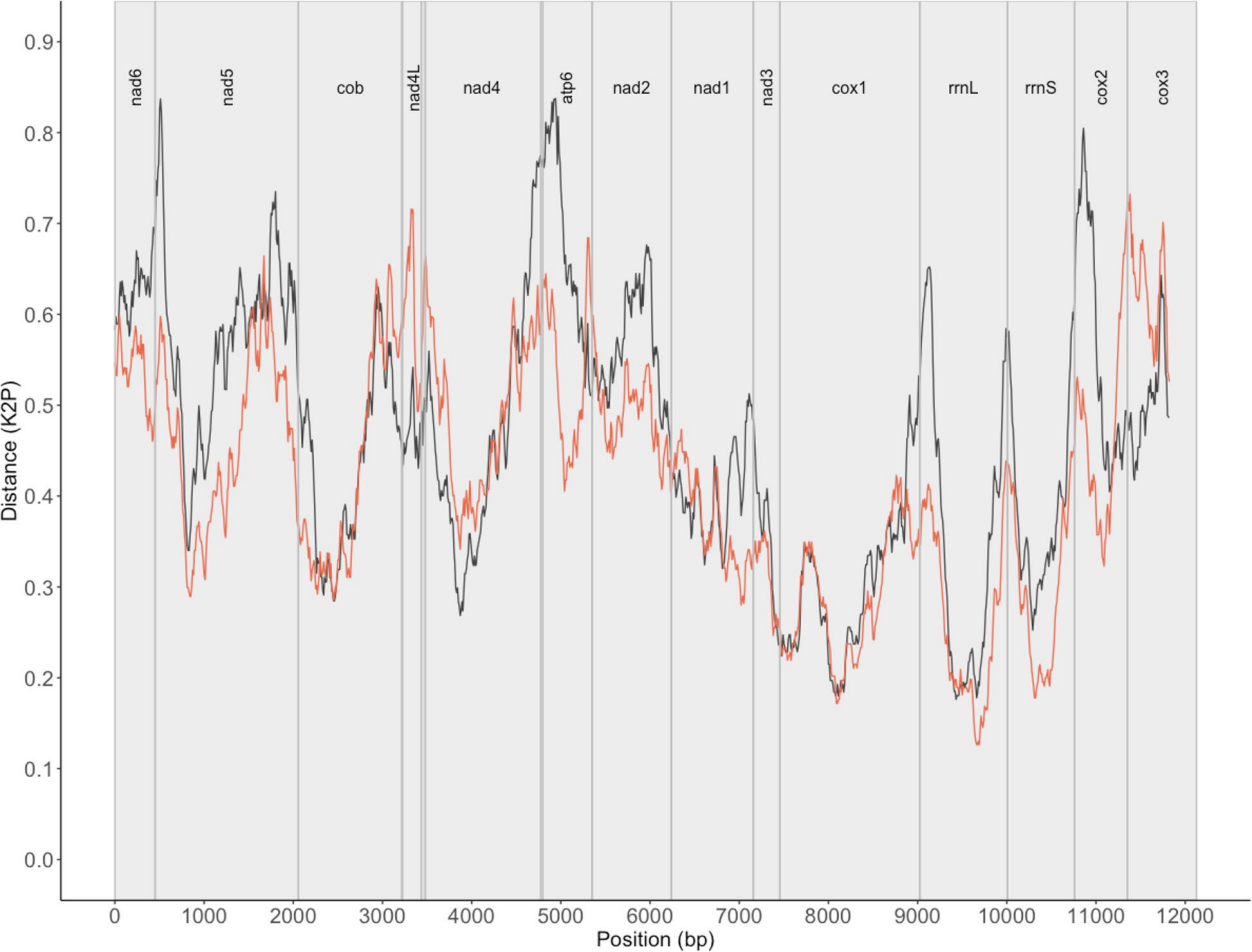
Interspecific comparison between *Eudiplozoon nipponicum* and selected diplozoids. Sliding window analyses (window size 300 bp, step size 10 bp) across the alignment of mitochondrial PCGs and rRNA regions show a pairwise comparison between *E. nipponicum* (present study: OQ331573) and representatives of different diplozoid genera, namely *Sindiplozoon* sp. (MG458326.1, the black line) and *Paradiplozoon yunnanensis* (NC_062047.1, the orange line). The lines show K2P distance, with gene boundaries indicated above the graph

A pairwise comparison of all four newly assembled *E. nipponicum* mitochondrial genomes (from this study) revealed a similarity between 99.82% and 99.93% with 46 SNPs across 13,772 bp of coding regions (excluding noncoding and repeat regions) and 99.97% in *cox1* region (1,572 bp). Intraspecific comparison between publicly available *E. nipponicum* mitochondrial genomes and the circular mitochondrial genome from this study revealed a 19.14% divergence across PCGs and rRNA regions (11,878 bp) compared to the *E. nipponicum* specimen collected from *C. auratus* (NCBI GenBank accession number MG458328.1), and a 5.56% variation in comparison with *E. nipponicum* collected from the unidentified cyprinid fish host (NCBI GenBank accession number NC_061193.1). A comparison of the *cox1* barcoding region (1,572 bp) revealed a similar level of divergence, namely 15.84% compared to MG458328.1 and 5.27% compared to NC_061193.1. Comparison between the species of genus *Paradiplozoon* shows differentiation ranging from 14.32% to 21.26% for PCGs and rRNA regions (12,010 bp) and from 10.74% to 15.19% in the *cox1* region (1,567 bp). Sliding window analyses revealed a substantial difference between the PCGs in the level of differentiation in all datasets, with *nad4* and *nad2* being the most variable regions on the intralineage level of *Eudiplozoon* spp. (Fig. 4) and *nad5*, *atp6* and *cox2* between representatives of different diplozoid genera (Fig. 5).

## Discussion

Monogeneans are probably the most diverse group of parasites within the Neodermata superclass. They are also the most host-specific parasites among flatworms [11, 12, 62]. High host-specificity correlates with a variety of unique morphological characteristics of their attachment organs, but a reliable classification of individual monogenean species cannot be established based solely on these morphological marks. This corresponds to a high number of publicly available monogenean molecular markers (e.g., sequences of 28S rRNA, *ITS1*, *cox1*, entire mitochondrial genomes) currently used for species classification [63–65] and identification of genetic population structures [21–23]. Despite improved accessibility of modern sequencing methods, whole-genome sequencing projects targeting monogeneans are limited to the genomes of just three monogenean representatives other than the present one. In particular, available in public databases are the genomes of two members of subclass Monopisthocotylea, namely *Gyrodactylus bullatarudis* [66], a parasite of the guppy fish *Poecilia reticulata*, and *Gyrodactylus salaris* [17], a severe parasite of the Atlantic salmon *Salmo salar*, and polyopisthocotylean monogenean *P. xenopodis* [5], which infects the African clawed frog *Xenopus laevis*. In this study, we combined short and long-read technology in a hybrid de novo assembly approach to create the first *E. nipponicum* genome draft, the second only representative of Polyopisthocotylea, conducted an in-depth analysis of predicted genes, and present candidate sets of PCGs relevant for its unique biology.

To generate a good de novo whole-genome assembly (error-free and identifying individual chromosomes) with a proper annotation for a non-model eukaryotic organism is a challenging bioinformatic task [67]. The completeness and contiguity of assemblies relies mostly on a sufficient amount of high-quality input data and, ideally, the deployment of a combination of different sequencing approaches, typically of the low error rate Illumina short reads with ONT or Pacific Biosciences long reads. But the quality and quantity of input data are merely two of numerous important factors in a puzzle that includes the complexity of the analysed genome, computational requirements, and applied software pipelines. In general, diploid helminth genomes are not considered highly complex [4]. Still, as technologies improve, long reads are essential to the ability to span across long repetitive regions. The current study presents the first ever ONT data for monogeneans. But it seems that the 7.72 Gbp generated by ONT, distributed in 2,287,049 trimmed and filtered relatively short reads (N50 = 8.23 kbp) (Table 2) was insufficient to fully resolve the highly repetitive genome of *E. nipponicum*, estimated to amount to a total of ~ 1.64 Gbp. It represents only ~ 4.70 times the sequencing depth of the genome size, whereby the processed Illumina short reads represent ~ 28.64 times the sequencing depth.

The predicted size of *E. nipponicum* genome (~ 1.64 Gbp) is almost three times larger than that of another polyopisthocotylean representative, *P. xenopodis* (617.34 Mbp) [5], and significantly (up to 25 times) larger than the size of genomes of monopisthocotyleans *G. bullatarudis* (84.34 Mbp) [66] and *G. salaris* (67.38 Mbp) [17]. Although the assembly draft did not reach the entire estimated genome size and did not resolve the chromosome level (2*n* = 14) [37], one can state that *E. nipponicum* has one of the largest genomes among parasitic helminths studied to date, currently surpassed only by genome size of the fluke *Dicrocoelium dendriticum* (1.89 Gbp, NCBI BioProject accession number PRJEB44434). *Eudiplozoon nipponicum* genome size exceeds the genome size of the human tapeworm *Spirometra erinaceieuropaei* (1.26 Gbp) [68] or the liver fluke *Fasciola hepatica* (1.20 Gbp, NCBI BioProject accession number PRJEB25283, updated genome draft originally published by Cwiklinski et al. [69]). The enormous size of *E. nipponicum* genome does not correlate with the chromosome number: for instance, the liver fluke *Clonorchis sinensis* with genome size 558.12 Mbp [70] has 28 pairs of chromosomes [71]. It seems that it should rather be ascribed to the high proportion of repetitive and low-complexity regions, which jointly amount to ~ 64% of length of the assembled genome. A high representation of repetitive regions has also been observed in the genome of *P. xenopodis* (52.64%), while in the genomes of two monopisthocotyleans, *G. bullatarudis* and *G. salaris*, it was notably lower (40.03% and 25.68%, respectively).

Differences between the two subclasses of Monogenea (Monopisthocotylea and Polyopisthocotylea) pertain not to the content of repetitive regions but for instance the number of predicted PCGs. Higher numbers of *E. nipponicum* and *P. xenopodis* PCGs (33,031 and 37,906) contrast with the numbers of PCGs in the smaller polyopisthocotylean genomes of *G. bullatarudis* and *G. salaris* (10,749 and 15,436, respectively). Unfortunately, the existing assembly of *P. xenopodis* genome is relatively fragmented (N50 = 2.9 kbp), which is why calculation of an average gene model may be biased. An average gene is 1.0 kbp long and contains two exons and one intron, with average exon and intron sizes of 312 bp and 537 bp, respectively. In contrast, the *E. nipponicum* 6.6 kbp gene includes three exons (279 bp) and two introns with an average size of 3 kbp. Monopisthocotylean gene models differ in their gene length (*G. bullatarudis* 4.7 kbp and *G. salaris* 2.7 kbp), the numbers of exons (six and four) and introns (four and three), and their typical intron length (769 bp and 659 bp), while their exons lengths are similar to *E. nipponicum* (288 bp and 289 bp). GC content in monogenean genomes ranges between 31.3% and 37.7%.

Hematophagy is a successful life strategy adopted independently by numerous multicellular parasites. Still, there are certain challenges associated with the blood diet on a molecular level which are likely to require specific adaptations, such as inhibition of haemostasis by anticoagulant factors [72], avoidance or blocking of host immunity by inhibition or modulation of immune mechanisms (such as complement cascade, phagocytosis, oxidative burst, or inflammation [73]), and efficient digestion of blood proteins. In these complex processes, peptidases occupy important positions; they are often organised in functional enzymatic cascades and regulated by specific endogenous inhibitors. Peptidases and their inhibitors play a crucial role in the pathogenicity of helminth parasites. They perform essential functions in a broad range of physiological processes, such as protein metabolism, feeding, anticoagulation, digestion, regulation of development, immune evasion, and reproduction, and they have been intensively studied and repeatedly reviewed [74–77]. Moreover, peptidases can be used to reveal both micro- and macroevolutionary changes during the evolution of parasites [18, 66]. Majority of identified individual peptidases (*n* = 54) belong to peptidases with serine catalytic type, specifically to the S1 family (chymotrypsin family, PA clan) and S1A subfamily (chymotrypsin A). These peptidases are involved in a broad range of biological processes, such as metabolism, digestion, regulation of development, and fertilisation [76]. Other member-rich peptidase families are, for example, the threonine T1 family (proteasome family, PB clan), which includes all identified threonine peptidases (*n* = 44) and whose size reflects the intensive protein turnover in the parasite’s metabolism, but also aspartic peptidases from the A1 family (pepsin family, AA clan) and A1A subfamily (*n* = 27), cysteine papain-like peptidases (C1 family, CA clan, C1A subfamily, *n* = 23), and the S33 family of serine peptidases (SC clan, prolyl aminopeptidase type, *n* = 20), which ensure critical biological processes and regulations. Aside from the previously characterised *E. nipponicum* peptidases (cathepsins B, L1, and L3 [30]), we have also investigated other cathepsins. The full spectrum of *E. nipponicum* cathepsins consists of cysteine cathepsin C (dipeptidyl peptidase I), which has been shown to be involved in the degradation of haemoglobin and peptides in the blood flukes *S. mansoni* and *F. hepatica* [74, 78], cathepsin D (lysosomal aspartic endopeptidase), which most likely plays a role in the reproduction and nutrition of the fluke *C. sinensis* [79], cathepsin K (lysosomal cysteine protease), whose function in helminths is unknown but elsewhere it acts in osteoblasts during bone remodelling [80], and finally, ribosomal proteinase cathepsin R. Among annotated peptidase inhibitors, we have identified for instance the serpinI2 (pancipin) inhibitor of serine peptidases, which belongs to the I4 family (ID clan, alpha-1-peptidase inhibitors), and two other inhibitors (thrombin inhibitor bothrojaracin and anticoagulant peptide haemadin), which are potentially involved in host–parasite interaction, specifically with the prevention of coagulation during blood feeding. Haemadin belongs to the I14 (hirudin type) family (IM clan) of inhibitors with well-known regulating functions during blood intake [81]. Bothrojaracin belongs to the I63 family (JB clan, pro-eosinophil major basic proteins), the third most abundant family by the number of protein molecules it contains (*n* = 4); it is known that by inhibiting thrombin, it prevents the formation of blood clots [82].

Tapeworms and flukes lack the ability to synthesise fatty acids de novo, at least in the adult stage [83, 84]. It is believed that this is a common characteristic of all parasitic platyhelminths. In *G. salaris* genome, only a gene for acetyl-CoA carboxylase has been identified [17]. *Eudiplozoon nipponicum* shares this general property but we have identified among the predicted proteins five enzymes involved in the biosynthesis of fatty acids (Additional file 4: Figure S1). Even so, the fatty-acid synthesis pathway of *E. nipponicum* is not complete and the parasite therefore cannot synthesise fatty acids de novo*.*

Haem is an iron-containing prosthetic group indispensable for the functioning of various proteins [85]. Following a previous transcriptome analysis [24], we can now finally confirm that *E. nipponicum*, like other hematophagous parasites, cannot synthesise haem de novo and solely depends on the host’s blood meal. Unlike other proteins involved in the haem synthesis cascade, gene-encoding 5-aminolevulinic acid synthase (ALAS) is not present in this genome assembly. Among functionally important molecules in the *E. nipponicum* biology, we have observed four proteins of metalloprotease endothelin-converting enzyme 1 (ECE1, M13 family), which is a highly expressed transcript in immature *S. mansoni* eggs [86]. ECE1 participates in the disruption of blood vessel wall and helps the eggs penetrate into the intestine and be excreted out of the host body [87]. In *E. nipponicum* biology, ECE1 should play a role during feeding and in the disruption of capillary walls, but the presence of ECE1 was confirmed only in the transcriptome, not in the secretome of the adult worms [24]. The distribution of plentiful Pfam domains in predicted *E. nipponicum* proteins (Additional file 3: Table S3) revealed 148 proteins with a protein kinase domain (PF00069). Eukaryotic protein kinases play a central role in many signal transductions via complex networks and they are viewed as a promising drug target for curing schistosomiasis [88, 89]. 122 proteins have RNA recognition motif domains (PF00076 and PF13893), which are characteristic for the RNA-binding proteins and generally abundant in parasitic helminths [90–93]. The third most represented domain is the zinc finger domain (C2H2 type, PF00096), represented in 113 proteins. Proteins with the zinc finger domain are involved in many essential processes and act as DNA/RNA binding proteins and transcription factors in the blood fluke *S. mansoni* [94]. Pfam domain PF00188 (CAP, cysteine-rich secretory protein family) was determined in 53 proteins. CAP proteins encoded in helminth genomes are believed to have immune-modulatory functions, which makes them attractive targets for vaccine or drug development [95]. Additionally, proteins with the CAP domain are the most abundant group in the excretory–secretory products of the human hookworm *Necator americanus* [96]. In *E. nipponicum* transcriptome, 18 proteins contain this domain with transcription power 509.78 TPM (transcripts per million) (TPM/transcript ratio: 28.32), but they were not detected in the secretome by mass spectrometry [24].

Researchers currently recognise 53 monogenean families [97]. Monogeneans are generally studied mainly in terms of phylogenetic classification, but interrelationships of monogenean taxa are not completely resolved despite intensive investigation of their morphology and numerous molecular phylogenetic studies. Previous studies had produced two mitochondrial genomes relevant to the genus *Eudiplozoon*. The first mitochondrial genome comes from the unspecified *Eudiplozoon* sp. (East China origin; fish host *Carassius auratus*; NCBI GenBank accession number MG458328.1 [35],) the second from *E. nipponicum* of unknown origin and host (NCBI GenBank accession number NC_061193.1; unpublished). Since it has been proposed that diplozoons infecting different cyprinid hosts should be classified as separate species, we have assembled a new mitochondrial genome (Czech Republic origin; fish host common carp) to further analyse the level of diversification related to host species origin. Despite minor morphological differences, comparison across mitochondrial genomes support the previously proposed existence of at least two distinct species of *Eudiplozoon*, which infect *C. carpio* and *Carassius* spp. [35, 36]. This is in line with previous hypotheses: It has been suggested that in monogeneans, the barcoding gap for *cox1* region should be at most 14.5%, but Vanhove et al. [98] report up to 15.84% differences across the entire *cox1* region. On top of that, certain differences related to the host species in the *ITS2* region also support differentiation on the level of species. Nishira and Urable [36] explicitly suggest the existence of two distinct species. On the other hand, since another study [36] found no clear differentiation in the *cox1* region according to the host species (*Carassius* spp. versus *C. carpio*) and given that the level of intraspecific variability in the mitochondrial genome in other monogenean lineages is high [99], future studies should further investigate the transect of geographical distribution of *Eudiplozoon* spp. to verify host specificity to *Carassius* spp. and *C. carpio*. Several instances of rearrangement of tRNA regions between diplozoid lineages are in line with the general consensus regarding a high variability of mitochondrial genome architecture in flatworms [100, 101]. The level of genetic distance within and between lineages differs across the PCGs, as reported in other monogenean families [100].

## Conclusions

Available comprehensive omics resources for monogenean parasites are, despite their clear ecological and economic importance, insufficient. Several recent studies have investigated molecular characteristics of these parasites (description of functionally important protein molecules), but majority of research in this area still focuses on monogenean morphology and phylogeny. We have used a hybrid de novo assembly method to create the first draft of nuclear genome of *E. nipponicum*. In this study, we thus present the largest monogenean genome and one of the largest genomes of parasitic helminths described as yet. This step was followed by a functional examination of those protein molecules, which are likely to play a key role in the host–parasite interaction and manipulation at a macromolecular level. We used the newly assembled mitochondrial genome variants to examine the intra- and interspecific differentiation of the family Diplozoidae and resolve the species status of *E. nipponicum*. The genome draft and mitogenome *E. nipponicum* presented here form a significant contribution to monogenean research and can serve as an essential source of information for further studies, but further thorough omics research is needed to better understand the nature of these parasites.

## Methods

### Parasite material

Living *E. nipponicum* adult worms were gradually collected during several summer and autumn periods in cooperation with local commercial fisheries (Rybářství Třeboň, Plc., Třeboň, Czech Republic) from naturally infected carps (*C. carpio*) bred in ponds in the south-western part of the Czech Republic (Třeboň Region). Severed heads of slaughtered fish were transported to the parasitological laboratory at the Faculty of Science, Masaryk University, where the gills had undergone a standard parasitological autopsy. Collected worms were thoroughly washed in 10 mM PBS buffer and stored in absolute ethanol at 4 °C.

### DNA extraction, library preparation, sequencing

Eight groups of worms (each consisting of five *E. nipponicum* adults) were used for DNA extraction and subsequent preparation of eight sequencing libraries: five Illumina libraries and three Oxford Nanopore Technologies libraries. In total, we have thus analysed 40 *E. nipponicum* individuals.

For Illumina sequencing, each group of five worms was first mechanically homogenised and then the DNA was extracted by DNeasy Blood and Tissue Kits (Qiagen) according to the manufacturer’s instructions. DNA concentration was quantified both spectrophotometrically (NanoDrop 8000, Thermo Fisher Scientific) and fluorometrically (Qubit 2.0, Life Technologies), and integrity verified using 2100 BioAnalyzer (Agilent Technologies). Library preparation and sequencing were carried out by the EMBL Genomics Core Facility (Heidelberg, Germany) on HiSeq 2000 Illumina (short-insert paired-end sequencing: two libraries in 2 × 100 bp configuration and one library in 2 × 125 bp configuration, all using TruSeq DNA PCR-Free Library Prep Kit, Illumina) and by the Institute of Molecular Genetics of the Czech Academy of Sciences (Prague, Czech Republic) on MiSeq Illumina (two libraries using short-insert paired-end sequencing, 2 × 251 bp configuration, performed with NEBNext Ultra DNA Library Prep Kit for Illumina, New England Biolabs).

The three remaining libraries were prepared for long-read sequencing on a MinION sequencer (ONT). High-molecular-weight DNA was extracted from three groups of worms using Genomic-tip 20/G (Qiagen) according to the manual, with one modification, namely overnight incubation (55 °C) with Proteinase K (Qiagen). DNA amounts were quantified by NanoDrop 8000 and Qubit 2.0 and integrity evaluated using 4200 TapeStation (Agilent Technologies). Libraries were prepared by Ligation Sequencing Kit (SQK-LSK108) according to manufacturer’s instructions and sequenced on the MinION device on FLO-MIN106 flow cells (one flow cell for each library, runtime 48 h). Library preparation and sequencing were performed at the Core Facility Genomics of CEITEC (Masaryk University, Brno, Czech Republic).

### The processing of raw sequencing reads

The quality of Illumina raw paired-end sequencing reads (in FASTQ format) was evaluated using FastQC v. 0.11.9 [102]. Sequencing adaptors and low-quality nucleotides (Phred score threshold 25, sliding window 4) were trimmed using the Trimmomatic v. 0.39 tool [103]. Sequencing errors were corrected by Musket v. 1.1 [104] and corrected reads deduplicated by ParDRe v. 2.1.5 [105]. Initial filtering was performed using Bowtie2 v. 2.3.5.1 [106] (*end-to-end* and *fast* modes), which maps the processed reads on the genome of the fish host *C. carpio* (BioProject PRJNA352247, assembly ASM1834038v1), because that is the most likely source of contaminations. Reads which were mapped as proper pairs and, at the same time, had quality above 30 were discarded. All generated ONT FAST5 files were basecalled in one batch using Guppy v. 4.4.1 (available via the ONT community site [107]). The quality of ONT raw reads (in FASTQ format) was assessed by NanoPlot v. 1.33.1 [108]. Adapters, low-quality nucleotides (quality score threshold 7) and very short reads (under 200 bp) were removed by Porechop v. 0.2.4 [109] and NanoFilt v. 2.5.0 [108]. Potentially contaminating reads originating from the fish host were identified after mapping on *C. carpio* genome by Minimap2 v. 2.17 [110]. Reads that had alignment block length over 500 bp and, at the same time, map quality above 30 were removed.

### Genome size estimation

Processed and filtered Illumina reads were used for bioinformatic estimation of genome size by counting k-mer frequency prior to assembly. K-mer occurrences were calculated and summarised as histograms by Jellyfish v. 2.3.0 [111] for k-mer lengths 15 to 31 (step by two) according to a previously described tutorial [112] (without quality masking). The resulting histograms were subsequently processed in R v. 4.0.3 [113] and genome size calculated based on the peak position. Additionally, the genome size was experimentally estimated using the flow cytometry method: A small part of fresh *E. nipponicum* individual was gently homogenised using a razor blade in 2 ml of cold Otto I buffer [114]. The crude suspension of nuclei was then filtered through a 0.2 μm nylon sifter and 0.5 ml of standard suspension added (male human leucocytes; 1C = 3027.52 Mbp; value following human/*Vicia faba* cv ‘Inovec’ ratio estimated by Doležel [115]). Finally, Otto II buffer (1.5 ml) containing fluorochrome propidium iodide was mixed with a filtered suspension of the sample and standard to stain the nuclei. After incubation of the mixture (at least 20 min, room temperature, darkness), flow cytometry measurement was performed using cytometer CyFlow ML (Partec GmbH; equipped with 100 mW laser Cobold Samba). Each measurement involved 5,000 particles. Sample/standard ratios were calculated from the means of histograms showing the relative fluorescence of the sample and standard by FlowMax software (Partec). The average coefficient of variation of all measurements was 3.63%. Four replicate estimates were performed (on different days) using the tissue of different *E. nipponicum* individuals. Final genome size was calculated as the average of all measurements. Flow cytometry analysis was performed in collaboration with the Laboratory of Molecular Bioszstematics, working group Plant Biosystematics (Department of Botany and Zoology, Masaryk University, Brno, Czech Republic).

### De novo genome assembly

We tested different approaches to the construction of *E. nipponicum* draft genome. The best possible genome draft (in terms of the level of fragmentation and overall length and completeness of the assembly) was assembled using the following procedure: Initial genome assembly was performed using the processed and filtered ONT reads by de novo assembler Flye v. 2.8.3 [116] (default parameters with three polishing iterations and minimum overlap between reads 1,000). This yielded a basic and long assembly draft. As the second assembler, we used the hybrid assembler MaSuRCA v. 4.0.3 [117], which took the processed and filtered ONT reads and merged them with Illumina unprocessed raw reads (according to documentation). MaSuRCA was run with default parameters, except for *JF_SIZE* being set to 20,000,000,000. Finally, both produced assemblies were merged by Flye v. 2.8.3 (*subassemblies* mode, 10 polishing iterations, minimum overlap between reads 5,000).

The resulting assembly was then repeatedly further filtered to remove possible contaminations. Using Minimap2 v. 2.24, all contigs were aligned on genomes of (a) the fish host *C. carpio* (BioProject PRJNA352247, assembly ASM1834038v1), (b) human (BioProject PRJNA31257, assembly GRCh38.p13), and (c) bacteria (all genomes deposited in NCBI RefSeq database, release 96 [118]). Contigs with alignment block length of over 3,000 bp and map quality above 40 were removed. Additionally, the filtered contigs were polished with processed and filtered Illumina reads by the polishing tool POLCA [119] and scaffolded by SAMBA scaffolder using the processed and filtered ONT reads. POLCA and SAMBA tools are available as parts of the MaSuRCA v. 4.0.3. Quality of the final version of the assembly draft (as well as of all interim versions) was assessed by BUSCO v. 5.2.2 (Metazoa dataset, odb10, 954 searched groups) [120] and Quast v. 4.6.3 [121].

### Gene prediction and annotation

All in all, the prediction of genes and protein-coding regions in the genome included the following steps: Core eukaryotic PCGs were identified in the final assembly using CEGMA v. 2.5 [122] and BUSCO v. 3.0.2 (Metazoa dataset, odb9, 978 searched groups). The latter was run with the *optimize_augustus* option to train the AUGUSTUS v. 3.3.3 ab-initio gene predictor [123] in the process. PCGs identified by the CEGMA were used to train the SNAP v. 2006–07-28 ab-initio gene predictor [124]. Species-specific repeats were identified using RepeatModeler v. 1.0.10 [125]. RepeatMasker v. 4.0.7 [126] was then run to mask repetitive regions 1) using the de novo library identified in the previous step, and 2) using a prebuilt repeat library (RepBaseRepeatMaskerEdition-20181026) with species set to *eukaryota*. Ab-initio gene predictor Genemark-ES (*gmes_petap.pl*) v. 4.69_lic [127] was run on the repeat soft-masked genome. As protein evidence that would further inform downstream gene prediction, we concatenated the complete UniProt/Swiss-Prot protein database (release 2022_01) [128] and 33 available protein complements of parasitic flatworms downloaded from the NCBI GenBank [129] and WormBase ParaSite databases [2] (accessed 2 February 2022). To remove redundancy in the reference protein set, it was clustered at 98% similarity using CD-HIT v. 4.8.1 [130]. Further, gene prediction was performed in two passes: First, using MAKER2 v. 2.31.10 [131] on the repeat masked genome, based on the physical protein (see above) and transcriptome evidence for *E. nipponicum* (DDBJ/ENA/GenBank accession GFYM00000000) [24], and using the gene models obtained with the SNAP (see above). Gene models of the first MAKER pass (only genes with evidence score < 0.2) were used to retrain the AUGUSTUS and SNAP ab-initio predictors. In the second pass, MAKER2 was rerun combining all evidence (protein and transcriptome as above) and using AUGUSTUS, Genemark, and SNAP and their pre-trained models. The resulting set of gene predictions was functionally annotated using the annotation module of Funannotate v. 1.8.7 [132], which combined the results from InterProScan v. 5.48–83.0 [133] with a similarity search against databases UniProt/Swiss-Prot (release 2022_01), MEROPS (database of proteolytic enzymes and inhibitors, release 12.0 [59]), and Phobius [134] using search tool DIAMOND v. 2.0.7 [135] (BLASTp algorithm) and with a search against the complete eggNOG 5.0 database [40] conducted with the eggNOG-mapper (emapper.py) v. 1.0.3 [39]. The entire prediction and annotation process as described above was run through the Annocomba [136], which uses the Snakemake workflow management system [137]. Furthermore, to obtain an even more comprehensive overview of the characterised functional molecules, we have supplemented the Funanotate annotation with several additional and /or modified analyses: using InterProScan v. 5.60–92.0 with GO annotation, we searched databases CDD, FunFam, Gene3D, PANTHER, PRINTS, Pfam, ProSitePatterns, ProSiteProfiles, SFLD, SMART, SUPERFAMILY, TIGRFAM, MEROPS (latest release 12.4) [59], UniProt/UniRef100 protein database (release 2022_05) [128], as well as NCBI non-redundant (nr) protein database and NCBI nucleotide (nt) database (both updated to 12 December 2022) [129]. Homologous sequences were searched with e-value threshold 1e^−5^ using Diamond v. 2.0.15.153 [135] for protein sequences and NCBI BLAST + v. 2.12.0 [138] for nucleotide sequences (BLASTn algorithm). We retained only the best, i.e., most significant, homologue for each *E. nipponicum* query transcript or protein from each database.

### Mitochondrial genome reconstruction and annotation

The processed and filtered reads from Illumina libraries were used to assemble the mitochondrial genome of *E. nipponicum*. Previously published *Diplozoon nipponicum cox1* sequence (NCBI GenBank accession number AY009163.1 [139]) was used as a seed for the NOVOPlasty v. 4.2.1 assembler [140] with a k-mer length ranging from 21 to ⁠⁠37 (odd numbers only), read length of 130 bp, and insert size of 390 bp. From libraries A7KL0, A72DD, and C841DACXX, we have reconstructed three individual non-complete (uncircularised) mitochondrial genomes. Circularisation of the final reference mitochondrial genome was achieved by a combination of overlapped regions of the assembled contigs from the A7KL0 library and reads from the C4VFYACXX library. Overlapping contigs were aligned and trimmed by Geneious Prime v. 2022.2.2 (Biomatters). In total, we created four variants of the mitochondrial genome for intraspecific differentiation analysis. The final circularised mitochondrial genome was annotated using the MITOS WebServer (echinodermal genetic code) [141] combined with the tRNAscan-SE [142] and RNAfold [143] web servers to verify the correctness of tRNA-coding regions. Subsequently, correctness of annotation was verified using a visualisation of open reading frames and alignment to selected mitochondrial genomes of closely related polyopisthocotylean monogeneans in Geneious Prime v. 2022.2.2., namely *Eudiplozoon* sp. (NCBI GenBank accession number MG458328.1 [35]), *E. nipponicum* (NCBI GenBank accession number NC_061193.1; unpublished), *Sindiplozoon* sp. (NCBI GenBank accession number MG458326.1 [35]), *Paradiplozoon yunnanensis* (NCBI GenBank accession number NC_062047.1; unpublished), *Paradiplozoon opsariichthydis* (NCBI GenBank accession number MG458327.1 [35]), and *Paradiplozoon hemiculteri* (NCBI GenBank accession number MW316634.1; unpublished). The presence and boundaries of repeat regions were investigated with the Tandem Repeats Finder v. 4.09.1 [144] and the final version of the *E. nipponicum* reference mitochondrial genome was visualised using OGDRAW v. 1.3.1 web server [145].

### Mitochondrial differentiation analysis

Intra-lineage differentiation (uncorrected percentage of distance) was calculated between the four versions of mitochondrial genome sequences assembled in this study (compared mutually) and between the circularised reference mitochondrial genome from this study (Czech Republic origin; fish host common carp) and two publicly available genomes, namely *Eudiplozoon* sp. (East China origin; fish host *Carassius auratus*; NCBI GenBank accession number MG458328.1) and *E. nipponicum* (unknown origin and host; NCBI GenBank accession number NC_061193.1; unpublished). Differentiation was assessed across PCGs and rRNA regions in Geneious Prime v. 2022.2.2. To further evaluate inter- and intraspecific differentiation across PCGs and rRNA regions, we applied the sliding window approach (step size 100 bp, window size 300 bp) in R package spider v. 1.5.0 [146] (R v. 4.2.0), using K2P distances to compare the two abovementioned publicly availably *Eudiplozoon* representatives. To further evaluate interspecific differentiations on a generic level, we performed a sliding window analysis across PCGs and rRNA mitochondrial regions to compare the species of *Paradiplozoon* (*Paradiplozoon yunnanensis* – NC_062047.1, *Paradiplozoon opsariichthydis* – MG458327.1, *Paradiplozoon hemiculteri* – MW316634.1). The pairwise comparison across PCGs and rRNA mitochondrial regions between *E. nipponicum* (GenBank number OQ331573) from the present study, *Sindiplozoon* sp. (MG458326.1), and *Paradiplozoon yunnanensis* (NC_062047.1) enabled us to evaluate differentiation on the generic level. We applied the rule of minimum bases lacking gene annotation. Sequences were aligned using Clustal Omega v. 1.2.3 [147] with fast clustering algorithm in Geneious Prime v. 2022.2.2. The resulting plots were visualised in R v. 4.2.0 with packages ggplot2 v. 3.3.6, mdthemes v. 0.1.0, ggtext v. 0.1.1, and ggfittext v. 0.9.0.

## Supplementary Information

**Additional file 1: Table S1.** Spreadsheet with complete statistics about the final version of the *E. nipponicum* genome assembly, including intermediate steps.

**Additional file 2: Table S2.** Text file with the complete results from analysis of repetitive and low-complexity regions.

**Additional file 3: Table S3.** Spreadsheet representing and summarising all results from the annotation of *E. nipponicum* predicted proteins.

**Additional file 4: Figure S1. **Graphical presentation of KEGG pathway map ko00061 (Fatty acid biosynthesis) with identified enzymes (highlighted in green) [38].

**Additional file 5: Table S4.** Spreadsheet presenting the complete list of identified peptidase families with catalytic types and numbers of involved proteins.

**Additional file 6: Table S5.** Spreadsheet with characteristics and positions of individual genes and regions in the *E. nipponicum* mitochondrial genome.

## Data Availability

All sequence data and genome assembly were deposited in NCBI. The Whole Genome Shotgun project was deposited at DDBJ/ENA/GenBank under accession no. JAQBSW000000000 (NCBI BioProject PRJNA914201). Version described in this paper is no. JAQBSW010000000.1. Assembled mitogenomic sequences are available in the DDBJ/ENA/GenBank under accession numbers OQ331573 (complete circular and annotated mitochondrial genome combining overlapping regions derived from the assembled contigs from Illumina libraries A7KL0 and C4VFYACXX), OQ434073 (uncircularised genome obtained only from Illumina library A7KL0), OQ434071 (uncircularised genome obtained only from Illumina library A72DD), and OQ434072 (uncircularised genome obtained only from Illumina library C841DACXX). Raw sequence data were deposited in the NCBI SRA database under accession numbers SRR22826952 (ONT) and SRR22826953–SRR22826957 (Illumina).

## Abbreviations

aa: Amino acids
bp: Base pairs
ECE1: Endothelin-converting enzyme 1
GO: Gene ontology
ONT: Oxford Nanopore Technologies
PCGs: Protein-coding genes
TPM: Transcripts per million
