# Supplementary figures and images for: An insight into the functional genomics and species classification of Eudiplozoon nipponicum (Monogenea, Diplozoidae), a haematophagous parasite of the common carp Cyprinus carpio
Source: BMC Genomics. 2023 Jun 29;24:363. doi: 10.1186/s12864-023-09461-8 (PMC10308649; doi:10.1186/s12864-023-09461-8)

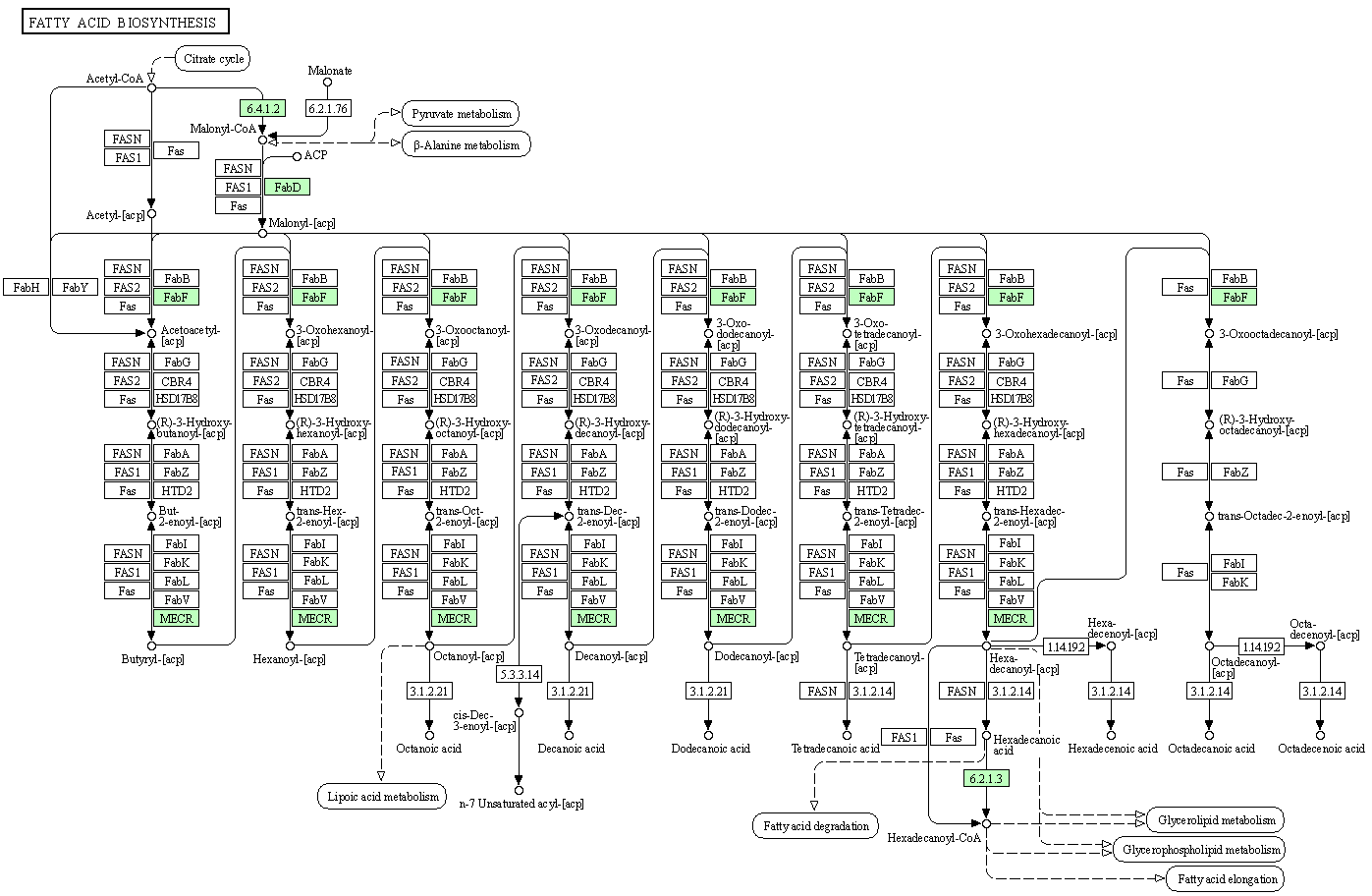

Supplement: Supplementary file 4 — Additional file 4: Figure S1. Graphical presentation of KEGG pathway map ko00061 (Fatty acid biosynthesis) with identified enzymes (highlighted in green) [38]. [file 12864_2023_9461_MOESM4_ESM.png]
